# Cultural activity at work: reciprocal associations with depressive symptoms in employees

**DOI:** 10.1007/s00420-019-01452-1

**Published:** 2019-06-11

**Authors:** Töres Theorell, Anna Nyberg

**Affiliations:** Stress Research Institute, University of Stockholm, Stockholm, Sweden

**Keywords:** Cultural activity, Gender, Education, Age, Depressive symptoms

## Abstract

**Purpose:**

Several studies have shown that cultural activities may promote health. There are also prospective population studies which show that regular participation in cultural activities could reduce morbidity and mortality. To what extent such associations could be applied to the work arena is not so well known, although findings in a few studies support the assumption that cultural activities organized from the work site might improve employee health. An important question discussed in the literature is the extent to which associations between cultural activity at work and employee mental health could be reversed, for instance, with depressive mood resulting in withdrawal from cultural activity at work (backwords) rather than the opposite (forwards). The present study addresses this question.

**Methods:**

Using a biennial national job survey with seven waves (SLOSH), we examined 2-year follow-up periods in 7193 men and 9313 women in the years 2006–2018. The question regarding cultural activity at work was examined prospectively (using multilevel structural equation modelling) both forwards and backwards in relation to a standardized score for depressive mood (SCL-CD_6_) in participants working at least 30% both at start and end of the 2-year period.

**Results:**

The analyses were made separately for men and women and with age and education level as confounders. The findings show that there are highly significant prospective relationships for both men and women in both directions concomitantly.

**Conclusions:**

Participation in cultural activity at work may protect employees from worsening depressive feelings, but depressive feelings may also inhibit participation in such activities.

## Introduction

Cultural activities can stimulate creativity and cohesiveness (Arnetz et al. 1983; Romanowska et al. 2011, 2016; Clift et al. 2007, 2008). Possible effects of cultural activities organized by the work place on employee health are, therefore, a potentially important subject for scientific exploration. We have previously published a study of the relationship between cultural activity at work and mental health among employees (Theorell et al. 2013). This previous study was based upon both cross-sectional and prospective observations of the Swedish working population. There were two prospective parts, one from 2006 to 2008 and one from 2008 to 2010. The cross-sectional analyses showed that there were significant correlations between cultural activity at work and low rate of emotional exhaustion. In addition, the follow-up from 2008 to 2010 showed a significant predictive value for cultural activities at work 2006 which was related to a favourable development of mental exhaustion from 2008 to 2010. The findings for depressive symptom development were also partly significant but weaker than for mental exhaustion.

On a more general arena, cultural activity in life as a whole, several prospective population studies have been published which show that lack of cultural activity in life is associated with poor health and vice versa (Bygren et al. 1996, 2009a; Clift et al. 2007, 2008, 2012). Konlaan et al. 2000, Tuisku et al. 2012, Väänänen et al. 2009). A recent large prospective population study (Løkken et al. 2008) in Norway has shown that cultural activity has a predictive value, however, differently for men (decreased mortality) and women (improved self-rated health). A Danish cross-sectional population study (Ekholm and Bonde 2008) has shown that “musicking” is associated with health but in different ways for men and women and also differently for professionals and amateurs. In addition, several dynamic studies have been published which show that the introduction of cultural activity is associated with improved health (Arnetz et al. 1983; Bygren et al. 2009b; Cohen 2009; Vaag et al. 2013, 2014)

One of the critical points in the discussion has been the extent to which the association between cultural activity and subsequent mental health is causal and the extent to which it could be reversed. A Swedish study (von Otter 2008) indicated that reversed causation (poor health causing diminished participation in cultural activities) could be a problem in the interpretation of associations. For instance, activity in a choir takes place on the condition that the voice is functioning—a choir singer stops singing when he/she gets voice problems. Therefore, the interpretation of temporal associations between cultural activity should ideally take into account that some of the observed relationships could be due to reversed causation—illness causing decreased cultural activity rather than the opposite.

In the present study, we have used the Swedish Longitudinal Occupational Survey of health (SLOSH) with its repeated waves of surveys 2006, 2008, 2010, 2012, 2014, 2016, and 2018. The occupationally active participants in the SLOSH have been analysed in multilevel structural equation modelling (MSEM). Our focus is on the development of depressive symptoms among working Swedish men and women in relation to cultural activity at work. The MSEM methodology allows the researcher to jointly analyze forwards and backwards, i.e., the extent to which cultural activity at work precedes improved mental state (in this case depressive symptoms), and at the same time, the extent to which depressive symptoms precede giving up cultural activity at work. In the multilevel data structure, the associations between the variables are estimated within individuals across time. All the successive 2-year intervals between pre- and post-observations have been summarized into a 2-year follow-up level in the equations. The two directions are taken into account concomitantly in the calculations of significance.

## Methods

### Study design

A longitudinal study design was applied in which lags and cross-lags between predictor and outcome variables were estimated with a 2-year time lag.

### Study sample

We used data from all waves (2006–2018) of the Swedish Longitudinal Occupational Survey of Health (SLOSH), a cohort derived from a nationally representative sample of the Swedish working population. SLOSH started in 2006 as a follow-up to the 2003 Swedish Work Environment Surveys (SWES) and participants from SWES 2005, 2007, 2009, and 2011 have been invited in later SLOSH follow-ups. Statistics Sweden collect data from the SLOSH cohort every second year. The respondents are invited to answer a self-completion questionnaire in two versions, one for those who work 30% or more of full time and another one for those who work less or not at all. More information on SLOSH can be found in the cohort profile (Magnusson Hanson et al. 2018). The cohort consists of 29,676 participants having responded to any SLOSH questionnaire at least once between 2006 and 2018. For the present study, we selected participants who had responded to the questionnaire for those who work 30% of full time or more in at least two consecutive waves (*n* = 16,506). The participants received written information about the study and, in accordance with Swedish regulation and practice, responding to and returning the survey indicated informed consent. The Regional Research Ethics Board in Stockholm approved the study. The funding sources had no role in the writing of the manuscript or in the decision to submit it for publication.

### Variables

Cultural activities were measured with the question “Are cultural events (films, plays, concerts, exhibitions) provided for the employees at your workplace?” with the response alternatives “never”, “once a year”, “once a month”, and “once a week or more often”. The variable was dichotomized into cultural events “not provided” or “provided” (the latter including once a year up to once a week or more often). Symptoms of depression were measured with the Symptom Checklist-Core Depression Scale (SCL-CD6), which is a 6-item subscale derived from the Symptom Checklist-90. The question “How much during the last week have you been troubled by…” is followed by six core symptoms of depression, namely “feeling blue/sad”, “feeling no interest in things”, “feeling low in energy”, “feeling that everything is an effort”, “worrying too much”, and “blaming yourself for various things”. The five response alternatives reach from “not at all” to “very much” and the depression index score ranges from 0 (lowest) to 24 (highest). Age was adjusted for in the categories ≥ 34, 35–44, 45–54, 55–64, and ≥65 years, and educational level in the categories 9 years of compulsory education, 2 years of upper secondary education, 3–4 years of upper secondary education, < 3 years of university education, and ≥ 3 years of university education. The analyses were stratified by gender.

### Analytical strategy

Autoregressive cross-lagged models within a multilevel structural equation modelling (MSEM) framework (Little 2013; Rabe-Hesketh et al. 2014) were fitted to the data, allowing to simultaneously address the reciprocal relationships between cultural activities at work (predictor variable) and symptoms of depression (outcome variable). In this multilevel approach, multiple measurement points (level 1) are nested within individuals (level 2) in a two-level structure that considers heterogeneity within individuals across time as well as between individuals. Symptoms of depression were measured as latent variables with six indicators. The fit of the measurement model as well as measurement invariance across time for the variable symptoms of depression were estimated. In gender-stratified analyses, we fitted structural equation models that allow for autoregressive as well as cross-lagged effects between cultural activities at work and symptoms of depression at each timepoint. The predictor variable cultural activities at work were measured at the first timepoint (*t *− 1) and symptoms of depression at the subsequent timepoint *t* years later (*t*) in data comprising the years 2006–2018. The simultaneous reverse association between symptoms of depression at the first timepoint and cultural activities at work 2 years later were also estimated. All paths in the models were adjusted for age and education; the indicators of the latent variable symptoms of depression were allowed to correlate between waves; all variables were treated as time-varying; and standardized model parameters were considered. Model fit was estimated with the root mean square error of approximation (RMSEA), the Tucker–Lewis index (TLI), the comparative fit index (CFI), and the standardized root mean square residual (SRMR), with model fits suggested to be acceptable when RMSEA ≤ 0.08, TLI ≥ 0.90, and CFI ≥ 0.90, and SRMR ≤ 0.08 (Little 2013). The multilevel SEM analyses were conducted in MPLUS version 8.

## Results

The distribution of the study variables is reported in Table 1 as means/standard deviations (depressive symptoms scores) and n/percentages of participants who reported cultural activity at work (yes) for all study years. Age and education-level distributions during the start year 2006 are also presented in the table. All the data are presented separately for men and women. In general, female participants reported more cultural activities at work than men, although the difference decreased towards the end of the observation period. As expected, women reported higher depressive symptoms scores than men. It is also observed that the highest percentages of participants reporting cultural activity at work were found in 2008 (49.4% for men and 55.2% for women). This culture peak year was followed during a 4-year period by a lowered level of cultural activity at work. After the cultural peak year, the depressive symptoms score decreased during the four following years both among men and women.

**Table 1:** Distributions/means and numbers of participants for the study variables in seven biennial waves of the survey study SLOSH 2006–2018

	Men (*n* = 7193)		Women (*n* = 9313)	
	Mean	Std	Mean	Std
Symptoms of depression 2006–2018				
2006	4.99	4.73	6.33	5.43
2008	5.01	4.94	5.96	5.36
2010	4.53	4.71	5.86	5.35
2012	3.96	4.40	5.03	5.04
2014	4.47	4.55	5.52	5.13
2016	4.37	4.64	5.40	5.15
2018	4.05	4.57	5.13	5.08

	*n*	%	*n*	%
Cultural activities (yes) 2006–2018				
2006	801	44.5	1022	48.7
2008	1816	49.4	2431	55.2
2010	1475	43.7	2028	46.4
2012	1224	42.9	1673	45.4
2014	2377	47.7	3297	48.7
2016	2273	44.4	3099	44.7
2018	1750	44.7	2474	45.0

	Men (*n* = 1889)		Women (*n* = 2300)	
	*n*	%	*n*	%
Age 2006				
≥ 34	279	14.8	378	16.4
35–44	482	25.5	572	24.9
45–54	551	29.2	753	32.7
55–64	555	29.4	592	25.7
65 years	22	1.2	5	0.2
Education 2006				
9 years compulsory education	332	17.8	313	13.7
2 years upper secondary	466	24.9	456	20.0
3–4 years upper secondary	429	23.0	431	18.9
< 3 years of university education	207	11.1	398	17.4
≥ 3 years of university education	434	23.2	685	30.0

Table 2 shows the results of the MSEM analyses. Age was highly significantly negatively associated with depression symptoms score—older subjects reported less depressive symptoms than younger. High education was negatively related to depression symptoms in men but not in women. Formulated in a different way, young men reported more depressive symptoms than other men. Similar observations were made for cultural activities—a high level of cultural activity at start was highly correlated with a high cultural level 2 years later. In men, high age was correlated with a high level of cultural activity at work, whereas no such finding was made for women. Both among men and women, a high level of education was associated with a high level of cultural activity at work.

**Table 2:** Standardised parameters (*B*), standard errors (SE), 95% confidence intervals (CI), and *p* values (*p*) among men (*n* = 7193) and women (*n* = 9313)

	Men (*n* = 7193)			Women (*n* = 9313)		
	*B* (SE)	95% CI	*p*	*B* (SE)	95% CI	*p*
Regression weights—DEP t						
DEP *t *− 1	0.637 (0.009)	0.619 to 0.655	0.000	0.595 (0.007)	0.580 to 0.609	0.000
CA *t *− 1	− 0.021 (0.006)	− 0.033 to − 0.009	0.001	− 0.016 (0.005)	− 0.026 to − 0.005	0.004
Age	− 0.089 (0.006)	− 0.101 to − 0.076	0.000	− 0.073 (0.005)	− 0.083 to − 0.063	0.000
Education	− 0.014 (0.013)	− 0.026 to − 0.003	0.013	0.007 (0.005)	− 0.004 to 0.017	0.215
Regression weights—CA t						
CA *t *− 1	0.422 (0.008)	0.406 to 0.439	0.000	0.401 (0.007)	0.386 to 0.415	0.000
DEP *t *− *t*	− 0.032 (0.008)	− 0.044 to − 0.014	0.000	− 0.018 (0.007)	− 0.024 to − 0.003	0.007
Age	0.020 (0.008)	0.005 to 0.036	0.009	− 0.100 (0.007)	− 0.024 to 0.003	0.141
Education	0.103 (0.008)	0.088 to 0.118	0.000	0.043 (0.007)	0.030 to 0.055	0.000

*CA* cultural activities, *DEP* symptoms of depression

**Fig. 1:**
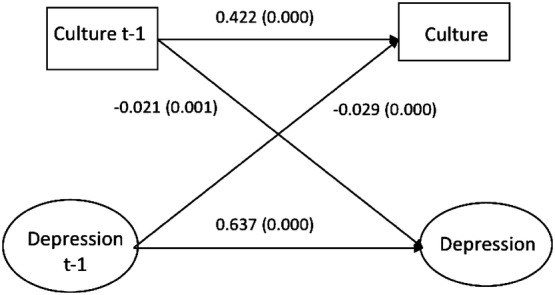
Structural paths between cultural activity at work and development of depressive feelings. Two-year intervals. Men

**Fig. 2:**
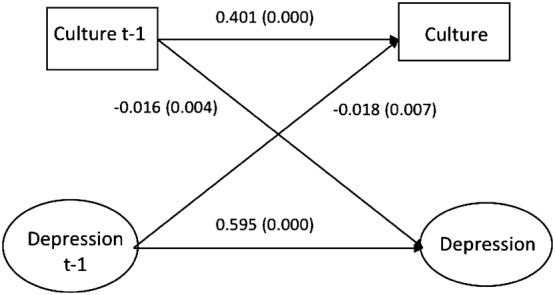
Structural paths between cultural activity at work and development of depressive feelings. Two-year intervals. Women

The measurement model for the latent variable symptoms of depression showed good fit statistics and there was no indication of measurement invariance over time. The standardized structural equation parameters of the cross-lagged associations between cultural activities and symptoms of depression are presented in Fig. 1 (men) and Fig. 2 (women). There were significant autoregressive paths between cultural activities *t *− 1 and *t* as well as between symptoms of depression *t *− 1 and *t* in both genders. The parameter for the association between cultural activities at the first timepoint (*t *− 1) and symptoms of depression 2 years later (*t*) was for men − 0.021 (*p* = 0.001) and for women − 0.016 (*p* = 0.004). This means that participants who reported having cultural activities at work at the first timepoint reported a 0.02 standard deviation decrease in symptoms of depression 2 years later compared to what they reported at the first timepoint. There was also a reverse association between the variables in both genders. The estimate for the association between symptoms of depression at the first timepoint (*t *− 1) and cultural activities 2 years later (*t*) was among men − 0.029 (*p* = 0.000) and among women − 0.018 (0.007). This gender stratified model presented good fit to the data: Chi-square (196) = 17415.887, *p* = 0.000, RMSEA 0.056, CFI 0.943, TLI 0.931, SRMR 0.034.

## Discussion

The results of this study indicate that there is indeed a forward association between cultural activity at work and a favourable development of depressive symptoms among employees. At the same time, there is also a backwards association—with depression comes decreased cultural activity.

The magnitude of the observed association between cultural activities at work and subsequent lowering of the prevalence of depressive feelings among employees is relatively small on the individual level. Translated to a population level, however, it is substantial. It should be pointed out that the cultural activities that the participants described mostly had a low intensity. According to the 45% of our participants that mentioned any cultural activity at work, the vast majority had only one such activity per year. Our findings could, therefore, be interpreted as an indication of the potential of cultural activities at work to reduce depressive feelings. More extensive well-planned cultural activities in work sites could potentially give rise to more substantial beneficial effects.

The mechanisms behind the forward association are unknown. Several factors may have a role. The question posed is of a general nature and limits “cultural” to the fine arts. It is also limited mainly to “consumption” of arts which may be reasonable in questions regarding the workplace. The more “active” the participants are in a cultural experience; however, the more likely it is that they will have an arousal effect of it (Theorell 2016). In other words, passive consumption may in general have weaker effects than active participation in cultural activities. Because of the question´s general formulation, we do not know anything about the relative mix of different kinds of arts; visual, music, and theatre. In rare instances, workplaces do organize choirs, music instrument groups, courses in painting and writing, or theatre performances in which employees are actors. However, we know very little about the extent of such activities in the work places. To the extent that employees do have joint experiences, for instance, of a theatre performance, there is also the possibility that they will discuss the contents in the experience and this may give rise to creative discussions. Employees may start talking to one another in unexpected ways and this may stimulate creativity and cohesiveness. This may illustrate that cohesiveness and being in control are stimulated by cultural activities at work.

Although the question about cultural activity at work included diverse cultural activities—attending theatre, movie, concert or going to exhibition—all these activities are documented as activities with a strong potential to stimulate cohesiveness in a group (Anderson et al. 2017; Teo et al. 2015; Boer and Abubakar 2014; Thomson et al. 2018) if the experiences are discussed in the group during or after the event. Thus, one of the main effects of these activities could be improved social support which may in itself protect against development of depressive symptoms (Åhlin et al. 2019).

A large number of factors may influence these findings. First of all, gender, age, and level of education could be confounders. We have adjusted the analyses for age and education and also performed the analyses separately for men and women. The SLOSH survey is constructed in such a way that new participants in the younger working ages have been added successively when the older subjects have left working life. The mean age has increased slightly among the participants (Magnusson Hanson et al. 2018).

Another strength of this study is that the statistical analyses are based on within-subject variations with the multilevel technique that we have applied. This means that most of the problem with potential unmeasured confounding is taken care of.

The analyses showed that separate path models for men and women provided a significantly better solution than a model including men and women together. This is due to the fact that the path from depressive feelings to decreased cultural activity at work is much stronger in men than in women. Accordingly, the tendency among employees with depressed feelings to not participate in cultural activities at work is much stronger in men than in women.

Some types of societal changes could also influence both cultural activities at work and symptoms of depression. One example is financial cycles. The cultural peak year 2008 coincided with the lowest unemployment rate (6.0%) in Sweden since the 1980s (Hörnqvist 2017). This year was a booming year in Swedish economy. However, in 2010, the unemployment rate had increased to 9% after which it decreased slowly to 7% in 2018. During these subsequent years, cultural activity in the work places was lower again. Thus, societal economy could possibly influence cultural activities in the worksites and at the same time stimulate optimism which means lowered depressive symptoms scores.

Several factors could mediate the association between cultural activity at work and decrease in depressive symptoms. For instance, an understanding boss might stimulate cultural activity at work (Theorell et al. 2013) and at the same time contribute to an improved psychosocial climate at work. It is hard to know how to interpret causality in such a complicated process.

Accordingly, there is empirical basis for both forwards and backwards associations between cultural activity at work and depressive symptoms. There are a few experimental studies (Bygren et al. 2009a; Romanowska et al. 2011; Vaag et al. 2014) which indicate that cultural activity at work may influence mental employee health favourably. Their findings are supported by our findings. That there is at the same time reversed findings is no surprise. However, rather than decreased participation in cultural activities at work, we would like to see that employees with depressive symptoms would increase their cultural participation!
